# Multi‐Frequency Electrocochleography Results in Fewer Drop Alarms During Cochlear Implant Insertion

**DOI:** 10.1002/lary.70592

**Published:** 2026-05-05

**Authors:** Mana Espahbodi, Ali Syed, Alex Tu, Michael D. Seidman, Christopher J. Danner, Kyle P. Allen, Loren J. Bartels, Syed F. Ahsan, Courtney C.J. Voelker, Nicholas L. Deep, Jacob B. Hunter, Rebecca C. Chiffer, Elias M. Michaelides, Mohamed Elrakhawy, Matthew W. Miller, Harrison W. Lin, Hamid R. Djalilian, Esther X. Vivas, Simon I. Angeli, Michael Hoa, H. Jeffrey Kim, Mark H. Widick, Katrina R. Stidham, Neil S. Patel, Richard K. Gurgel, Karl W. Doerfer, Kanthaiah Koka, Michael S. Harris

**Affiliations:** ^1^ Department of Otolaryngology—Head and Neck Surgery University of Utah Salt Lake City Utah USA; ^2^ Department of Otolaryngology & Communication Sciences Medical College of Wisconsin Milwaukee Wisconsin USA; ^3^ AdventHealth; University of Central Florida Orlando Florida USA; ^4^ Tampa Bay General Hospital Tampa Bay Florida USA; ^5^ Head & Neck Surgery Department Southern California Kaiser Permanente Medical Group Anaheim California USA; ^6^ Otology/Neurotology‐Lateral Skull Base Surgery Pacific Neuroscience Institute Los Angeles California USA; ^7^ Mayo Clinic Comprehensive Cancer Center Otolaryngology (ENT)/Head and Neck Surgery, Mayo Clinic Scottsdale Arizona USA; ^8^ Department of Otolaryngology Thomas Jefferson University Hospital Philadelphia Pennsylvania USA; ^9^ Department of Otolaryngology‐Head and Neck Surgery Rush University Medical Center Chicago Illinois USA; ^10^ Otolaryngology‐Head and Neck Surgery, Sanford Health Fargo North Dakota USA; ^11^ Department of Otolaryngology—Head and Neck Surgery University of California Irvine California USA; ^12^ Department of Otolaryngology Head and Neck Surgery Emory University Atlanta Georgia USA; ^13^ Department of Otolaryngology, UHealth Ear Institute University of Miami Miller School of Medicine Miami Florida USA; ^14^ Department of Otolaryngology—Head and Neck Surgery MedStar Georgetown University Hospital Washington DC USA; ^15^ ENT of South Florida Boca Raton Florida USA; ^16^ Department of Otolaryngology—Head and Neck Surgery Westchester Medical Center Valhalla New York USA; ^17^ Research and Technology, Advanced Bionics LLC Valencia California USA

**Keywords:** cochlear implantation, ECochG, multi‐frequency electrocochleography

## Abstract

**Objective:**

To evaluate intracochlear electrocochleography (ECochG) amplitude parameters during cochlear implantation (CI) using a novel multi‐frequency ECochG algorithm.

**Methods:**

A multi‐institutional, prospective cohort study was performed at 18 high‐volume CI centers. The inclusion criteria were adults with sensorineural hearing loss and audiometric thresholds of ≤ 90 dB hearing level at 500 Hz undergoing CI with Advanced Bionics (Valencia, CA) Ultra 3D devices between 2024 and 2025. ECochG recordings were performed with simultaneous multi‐frequency stimulation of four frequencies between 125 and 4000 Hz during cochlear implant insertion. Concurrent multi‐frequency recording allowed extraction of amplitude and phase of each frequency individually. Post hoc analysis was performed to determine the difference in the number of drop alarms between single‐ and multi‐frequency ECochG. An ECochG amplitude drop of 6 dB was defined as a drop alarm. Insertion track patterns were compared between single‐ and multi‐frequency ECochG.

**Results:**

One hundred ninety‐five ears were included. Mean number of drop alarms for the single‐frequency algorithm was 1.72 (95% CI: 1.52, 1.92; median 1) compared to 0.42 (95% CI: 0.31, 0.53; median 0) for multi‐frequency; *p* < 0.001. The number of Type C patterns (rise in amplitude during insertion followed by a drop) decreased with the multi‐frequency ECochG algorithm compared to the single‐frequency ECochG algorithm. The number of Type D patterns (no‐response) decreased, indicating that multi‐frequency ECochG generated more responses across the cochlea than single‐frequency ECochG.

**Conclusions:**

A novel multi‐frequency ECochG algorithm during CI is associated with fewer drop alarms and altered insertion track patterns, which may provide a more accurate assessment of the cochlear microenvironment.

**Level of Evidence:**

3.

## Graphical abstract

A novel multi‐frequency electrocochleography (ECochG) algorithm used during cochlear implant electrode insertion is associated with fewer drop alarms and an increase in optimal insertion track patterns. Multi‐frequency ECochG may provide a more accurate assessment of the cochlear microenvironment when compared to single‐frequency ECochG.

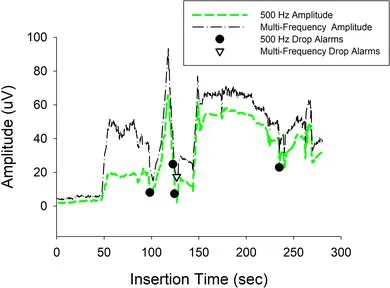

## 1 Introduction

Cochlear implantation (CI) is the standard of care treatment for patients with severe‐profound sensorineural hearing loss (SNHL) and poor speech recognition. Contemporary cochlear implant patients frequently have preserved low‐frequency hearing, with more severe hearing loss in the mid‐ and higher frequencies. One of the potential side effects of CI is damage to the microstructures of the cochlea and loss of residual hearing. Preservation of cochlear structures and residual hearing has been shown to correlate with better postoperative speech perception, offers the possibility of electro‐acoustic stimulation, and may facilitate future treatment options for SNHL [1, 2, 3, 4, 5]. Despite progress in ‘soft’ surgical technique and electrode design, variability persists in hearing preservation rates, limiting access to these well‐demonstrated benefits of preserved acoustic hearing [6].

**FIGURE 1:**
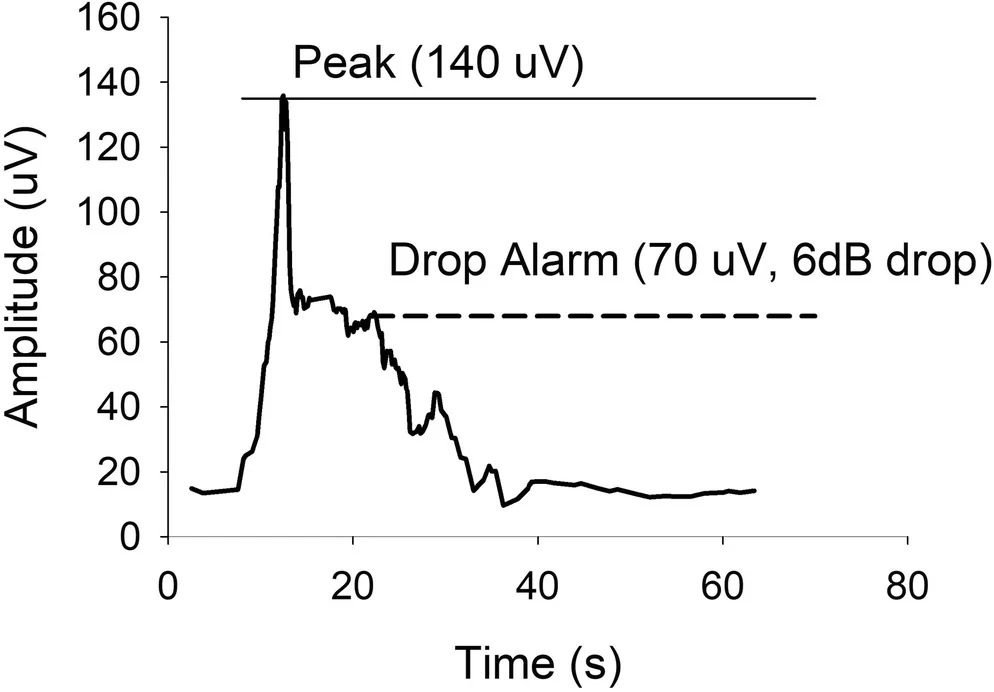
A representative example of a Type C single‐frequency ECochG pattern (characterized by an initial peak followed by a drop). A drop alarm was set at 6 dB (50% drop in amplitude from peak).

Intracochlear electrocochleography (ECochG) is an acoustically evoked electrical potential response from the cochlea that provides a live, objective measure of cochlear integrity and has been shown to aid in monitoring residual hearing and cochlear structure preservation during CI [2, 7, 8, 9, 10, 11] and to optimize electrode placement [12]. Traditional intracochlear ECochG algorithms are based on single‐frequency tone‐burst stimuli (250, 500 Hz, or any single frequency based on preoperative audiogram). In traditional commercially‐available ECochG, a single‐frequency stimulus at 90–115 dB hearing level (HL) is presented, and electrical potentials—including the cochlear microphonic (CM)—are generated by the sensory epithelium of the cochlea and the auditory nerve. The electrophysiological signal is measured using one of the cochlear implant electrode array contacts within the cochlea, sampled by the internal receiver/stimulator, and sent for processing and analysis. An auditory and visual display communicate changes in ECochG amplitude to the surgeon. Users can select a threshold to identify an event and notify the surgical team of the event as a “drop alarm.” A drop alarm based on a single‐frequency ECochG amplitude is exemplified in Figure 1 for a representative patient. Commercially available ECochG systems can present a single, user‐selected frequency between 125 and 4000 Hz to the patient's ear and monitor ECochG responses from the cochlea during electrode insertion.

A potential shortcoming of traditional ECochG algorithms is that all signal drops, chiefly reflecting the cochlear microphonic (CM), which indicates outer cochlear hair cell function, are treated as traumatic interactions. Saoji et al. showed that ECochG drops elicited by a single frequency may not translate to drops across all frequencies [13]. Additionally, an unknown proportion of CM drops may occur in the absence of trauma and may, alternatively, signify advancement of the recording electrode past the acoustic generator site in the cochlea, as highlighted by phase interactions [2, 12, 14, 15].

Novel multi‐frequency recorded ECochG algorithms, which include up to four simultaneously presented frequencies, have been developed to better differentiate traumatic from atraumatic insertions. This paper reports results from a multi‐institutional prospective cohort study that examined the multi‐frequency algorithm described by Babajanian et al. [16] Babajanian et al. selected four frequencies based on preoperative audiograms that varied across patients [16]; the current study utilized four frequencies between 125 and 4000 Hz for all subjects. The primary objective of the current study was to show that the number of drop alarms seen by multi‐frequency ECochG is significantly lower than single‐frequency ECochG, possibly representing a decrease in the number of false alarms (i.e., “false positives,” implying intracochlear trauma when there is not any) during cochlear implant electrode insertion. The secondary objective was to determine if multi‐frequency ECochG responses can be elicited in participants with greater hearing loss compared to single‐frequency ECochG.

## 2 Materials and Methods

### 2.1 Participants

Study participants were recruited from 18 high‐volume CI centers in the United States (US). Each participating CI center received approval from its Institutional Review Board (IRB) or the equivalent. All study procedures complied with the ethical standards of the Helsinki Declaration [17]. Prior to enrollment, all participants met their clinical institutional CI candidacy criteria and chose an Advanced Bionics (AB; Valencia, CA) Ultra 3D device with either a lateral wall SlimJ or a pre‐curved HiFocus MidScala (HFMS) electrode.

For study inclusion, participants were required to have unaided pure‐tone air‐conduction (AC) thresholds of ≤ 90 dB hearing level (HL) at 500 Hz and had to be at least 18 years old. Exclusion criteria were malformed cochlea, presence of myringotomy tubes, chronic otitis media, middle ear surgery, or temporal bone trauma.

### 2.2 Multi‐Frequency ECochG Acquisition

After administration of general anesthesia, and before the patient is prepped and draped for CI, an in‐ear earphone is inserted into the external ear canal of the ear to be implanted. The earphone connects to a plastic tube that allows the AIM system (AIM Advanced Bionics LLC, Valencia, CA, USA) to provide acoustic stimulation of the ear and take corresponding ECochG measurements. After placement of the receiver/stimulator and before electrode insertion, a Naída CI Q90 sound processor with an extended programming cable is used to connect the AIM system to the cochlear implant. The AIM system (AIM1.2R) is then used to perform multi‐frequency ECochG recording during electrode insertion.

AIM1.2R allows users to present multiple frequencies simultaneously as selected. AIM1.2R multi‐frequency ECochG responses are recorded without changing the standard of care procedures (without any multi‐frequency auditory or visual alarms provided to the surgeon) for cochlear implant electrode insertion. The default four frequencies of multi‐frequency stimuli used in this study were 125, 250, 500, and 1000 Hz, with default stimulus levels of 90, 95, 105, and 105 dB HL, respectively. A multi‐frequency stimulus is a single auditory stimulus composed of multiple frequency components that are distinct in the spectral (fast Fourier transform [FFT]) domain. A stimulus is generated by combining multiple distinct sinusoids. All four components are generated in such a way that all are non‐harmonic of each other and can be identified as four distinct peaks in FFT spectrum. The ECochG signal, when analyzed in FFT domain, gives access to the components (amplitude and phase) of each frequency. All the amplitudes can be combined into a multi‐frequency ECochG pattern. Multi‐frequency algorithms record amplitude and phase changes among all the four frequencies. This approach allowed for the simultaneous application of both algorithms in this study post hoc.

### 2.3 Outcome Measures

**FIGURE 2:**
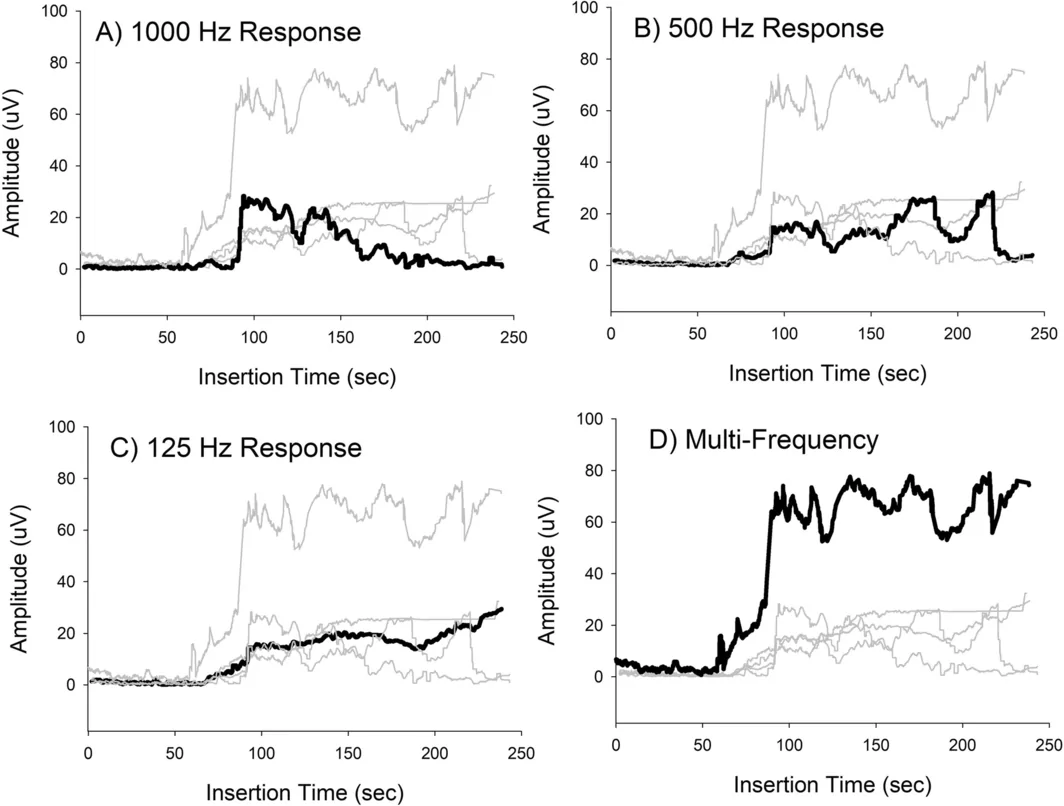
A representative example describing multi‐frequency ECochG (Panel D) and responses at each frequency (Panel A—1000 Hz, Panel B—500 Hz, Panel C—125 Hz; ECochG response line for that respective frequency is bolded in each panel). Both 1000 and 500 Hz exhibit sequential drops, whereas 125 Hz does not. A single frequency ECochG would have presented a drop alarm at 500 Hz response around 225 s. However, multi‐frequency ECochG (Panel D) does not demonstrate a drop and remains stable, meaning no drop alarm is triggered when multiple frequencies are considered.

**FIGURE 3:**
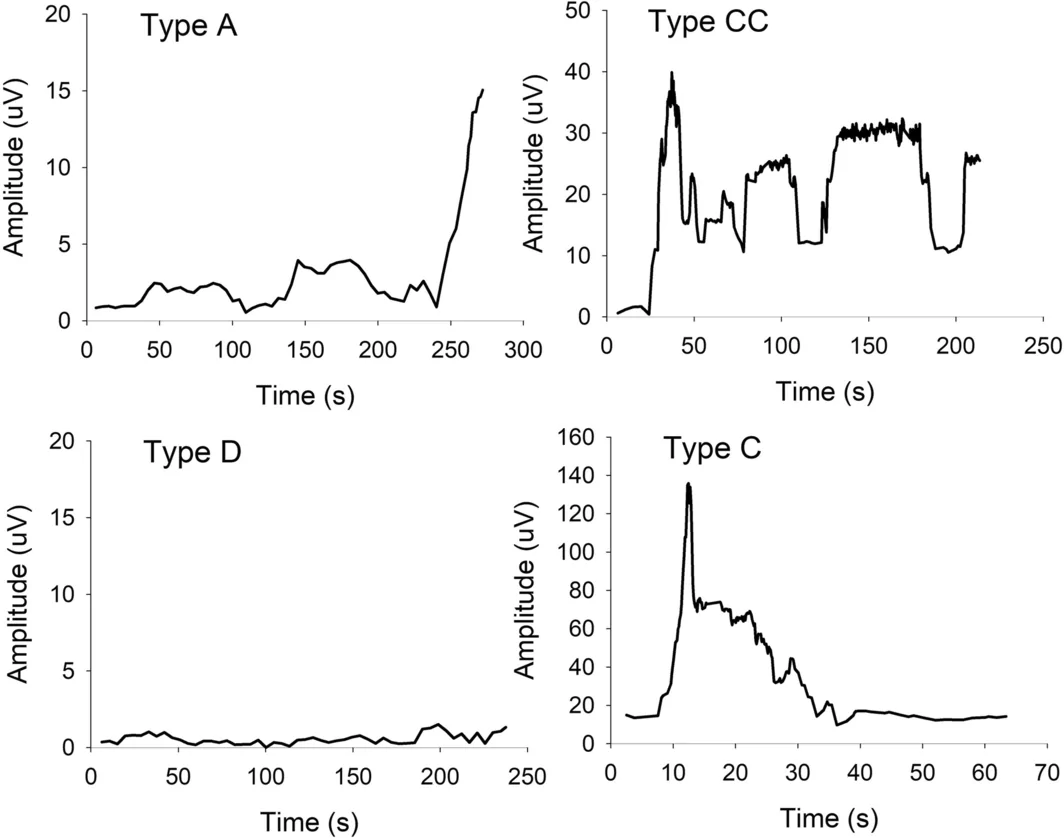
Four ECochG insertion track patterns were observed in this sample. The Type A pattern is characterized by an increase in amplitude from the start of insertion at the round window through completion of insertion without a signal drop (< 6 dB loss). The Type C pattern is defined by an amplitude at the start of insertion comparable to the amplitude after insertion, with amplitude maxima reached mid‐insertion. Type CC consists of an initial peak, followed by fluctuations then a recovery from the lowest drop amplitude. The Type D pattern is characterized by no response above the noise floor.

An ECochG amplitude drop of 6 dB was defined as a drop alarm. The multi‐frequency algorithm examines the amplitude and phase across multiple electrodes and triggers a drop alarm only when at least two frequencies simultaneously experience amplitude drops and significant phase changes [16]. Figure 2 illustrates a conceptual example of multi‐frequency ECochG recordings, showing individual responses at each frequency compared to the combined response across all four frequencies. Panels A, B, and C show ECochG waveforms at 1000, 500, and 125 Hz, respectively. Several distinct ECochG amplitude patterns observed during cochlear implant electrode insertion have previously been described (Harris et al. see also Koka et al. and Giardina et al. for alternative classification systems) [2, 12, 18]. The Type A pattern is characterized by a continued increase in ECochG amplitude from the start of insertion at the round window through the completion of insertion—the amplitude has to rise more than 6 dB from initial response [18]. The Type C pattern is defined by an amplitude at the start of insertion comparable to the amplitude at completion of insertion, with the maximum reached mid‐insertion and a precipitous drop in signal thereafter—the amplitude has to rise by more than 6 dB at mid‐insertion compared to the initial response, but has to drop more than 6 dB after reaching the peak amplitude and never recover. The Type D pattern is no response above the noise floor—the amplitude did not change more than 6 dB compared to the initial response. The Type CC pattern is characterized by an initial peak, followed by fluctuations, then a recovery from the lowest drop amplitude—the amplitude rises more than 6 dB followed by a drop of more than 6 dB and then a recovery to a final amplitude within 6 dB of the peak value [19]. Insertion track patterns were compared between 500 Hz, single‐frequency, ECochG, and multi‐frequency ECochG at four frequencies between 125 and 4000 Hz. Four representative insertion tracks are plotted in Figure 3.

In this study, during electrode insertion, multi‐frequency ECochG was collected, and post hoc analysis was conducted to compare the number of drop alarms generated by single‐frequency and multi‐frequency algorithms using intra‐participant data. Surgeons did not have auditory or visual access to feedback from multi‐frequency ECochG, however; access to the standard of care, single‐tone, auditory and visual feedback was available to surgeons and was utilized according to surgeon preference.

### 2.4 Statistical Analysis

Statistical analyses were performed using SPSS Statistics (Version 22; IBM Corp., Armonk, NY). *T*‐tests (Shapiro–Wilk) were used to compare means for normally distributed data. Non‐parametric Mann–Whitney U tests were performed to compare non‐normally distributed data, including the number of drop alarms between single‐frequency and multi‐frequency ECochG. Statistical significance for all tests was set at *p* ≤ 0.05.

## 3 Results

**FIGURE 4:**
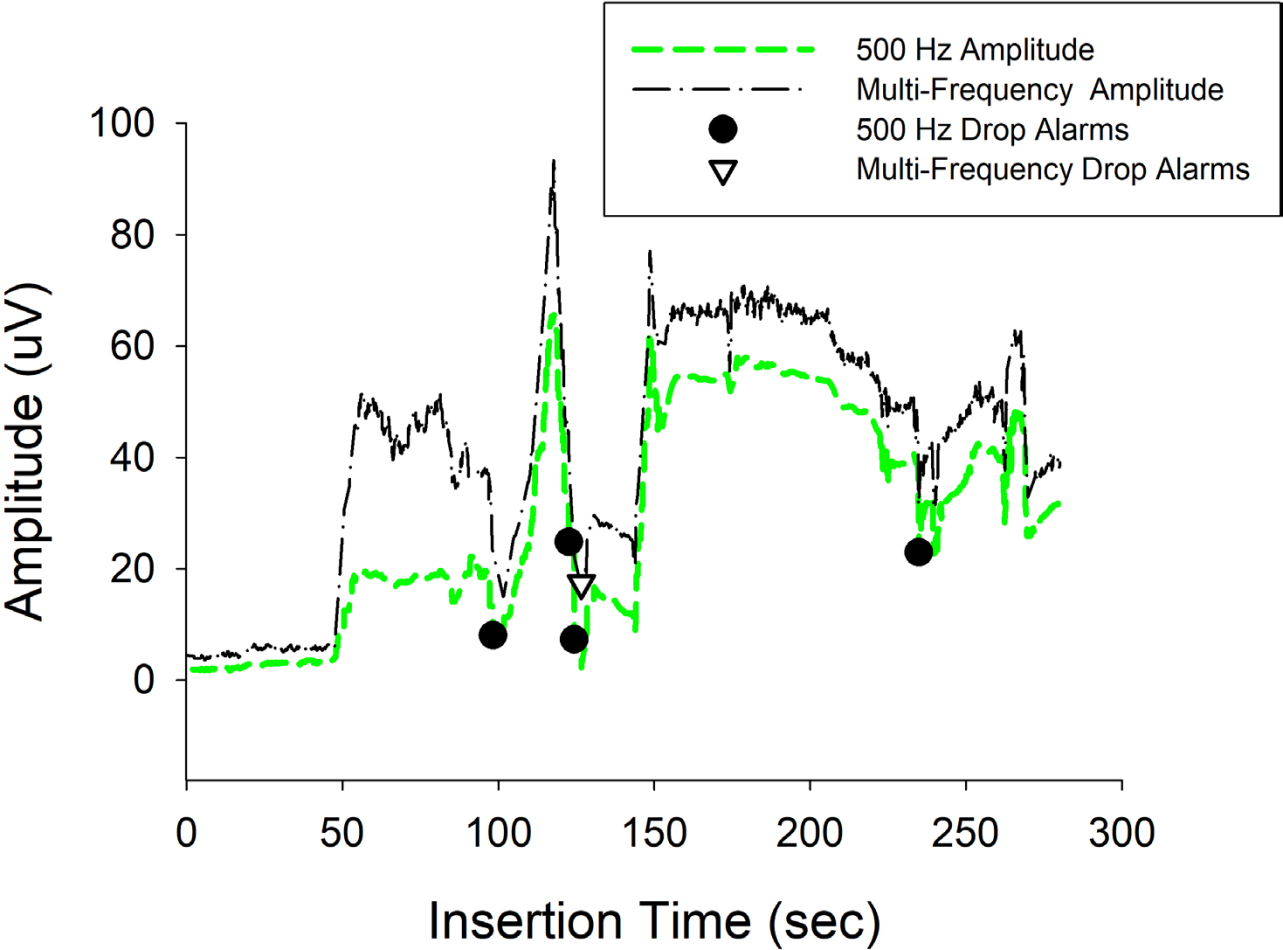
An example of an ECochG pattern tracing that results from the multi‐frequency algorithm compared to the single‐frequency (500 Hz) algorithm. The multi‐frequency pattern had one drop alarm, while the single‐frequency (500 Hz) pattern had four drop alarms. [Color figure can be viewed in the online issue, which is available at www.laryngoscope.com]

A total of 195 adult ears who received AB Ultra3D cochlear implants across 18 institutions were included in this study. Seventy‐five ears were implanted with Mid‐scala electrodes, and 120 with SlimJ electrodes. A representative example demonstrating differences in drop alarm detection between single‐frequency ECochG and multi‐frequency ECochG is shown in Figure 4. The graph presents data from the same insertion, plotting the single‐tone low‐frequency stimulus (500 Hz) ECochG amplitude and the multi‐frequency ECochG amplitude across four simultaneous frequencies between 125 and 4000 Hz. With a pre‐selected default drop alarm level of 6 dB (equivalent to an amplitude drop of 50%), four drop alarms were identified when using the 500 Hz ECochG amplitude alone. In comparison, when the amplitude and phase across four frequencies were utilized, there was only one drop alarm (Figure 4). Using the 500 Hz ECochG response, the insertion track pattern is consistent with a Type C (associated with poorer cochlear structural preservation) [18]. Whereas, using the multi‐frequency algorithmic approach, the waveform is consistent with a Type A (associated with better cochlear structural preservation) [18].

**FIGURE 5:**
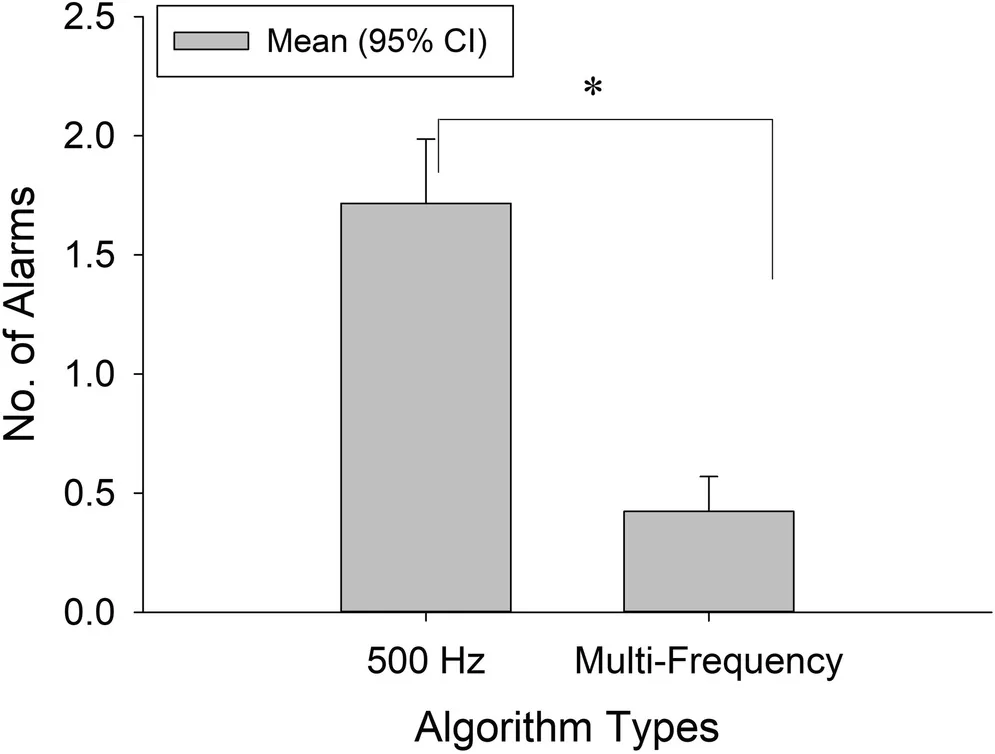
Comparison of drop alarms between single‐frequency (500 Hz) and multi‐frequency ECochG. The mean number of drop alarms for the single‐frequency algorithm was 1.72 (95% CI: 1.52, 1.92; median of 1), whereas the mean number of drop alarms for the multi‐frequency algorithm was 0.42 (95% CI: 0.31, 0.53; median of 0); *p* < 0.001*.

Figure 5 shows a comparison of the number of drop alarms between 500 Hz single‐frequency ECochG and multi‐frequency ECochG. A *t*‐test (Shapiro–Wilk) indicated that the data did not follow a normal distribution, and a non‐parametric Mann–Whitney U test was performed to compare the number of drop alarms between single‐frequency and multi‐frequency ECochG. The Mann–Whitney U test revealed a statistically significant difference between the groups (*U* = 947.000, *T* = 5133.000, *n*_1_ = *n*_2_ = 195, *p* < 0.001). The mean number of drop alarms for the single‐frequency algorithm was 1.72 (95% CI: 1.52, 1.92; median of 1), whereas the mean number of drop alarms for the multi‐frequency algorithm was 0.42 (95% CI: 0.31, 0.53; median of 0).

**FIGURE 6:**
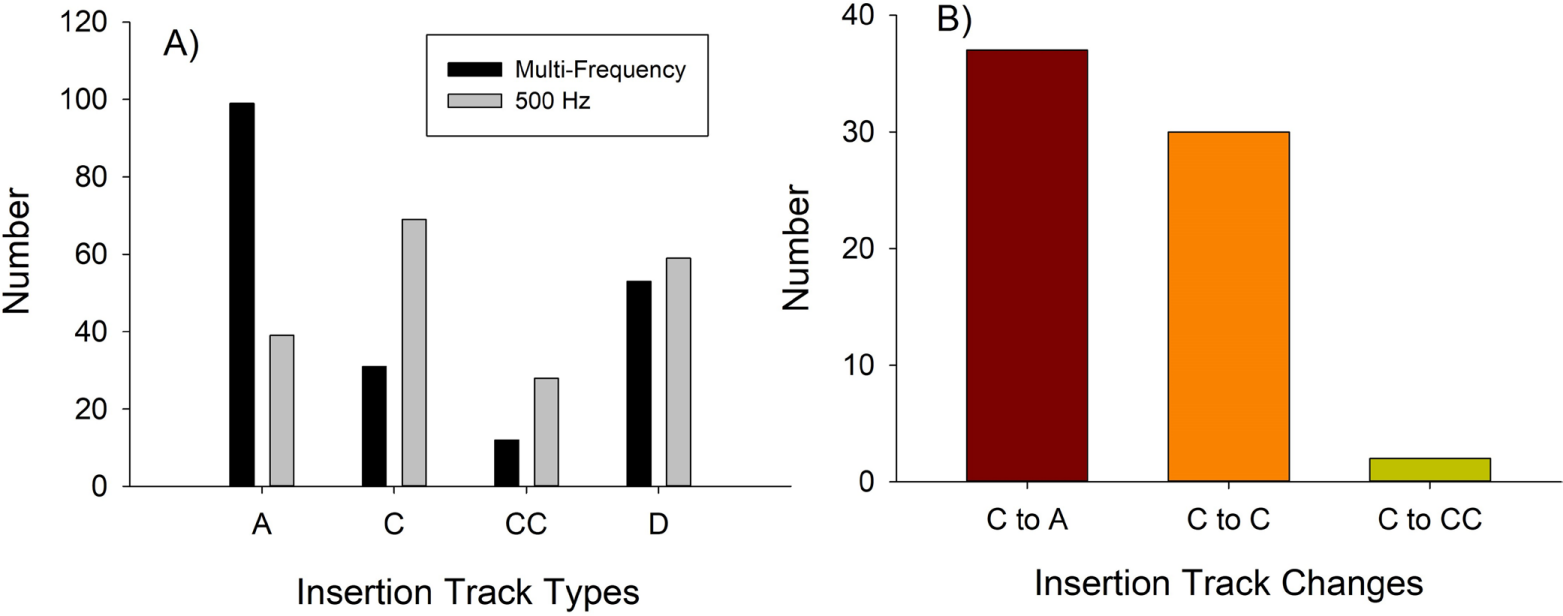
Panel A: Insertion track type comparison between single‐frequency (500 Hz) and multi‐frequency algorithms. Panel B: Insertion track changes from type C insertion track pattern to other patterns based on the change from the single‐frequency to multi‐frequency algorithm.[Color figure can be viewed in the online issue, which is available at www.laryngoscope.com]

Figure 6, panel A shows a comparison of insertion track types between single‐frequency (500 Hz) ECochG and multi‐frequency ECochG. Multi‐frequency algorithm‐based classification showed fewer Type C insertions compared to the single‐frequency algorithm. The multi‐frequency algorithm also showed an increase in Type A insertions compared to the single‐frequency algorithm, as single‐frequency drops may not always result in multi‐frequency drops. Panel B of Figure 6 shows the number of insertion pattern changes from Type C to other patterns when the multi‐frequency algorithm was compared to the single‐frequency approach. Out of 69 Type C patterns in the single‐frequency algorithm, 37 converted to Type A, 30 stayed at Type C, and two changed from Type C to CC when the multi‐frequency algorithm was utilized post hoc.

## 4 Discussion

This study showed that in a large sample of cochlear implant recipients, multi‐frequency ECochG was associated with significantly fewer drop alarms compared to single‐frequency ECochG when both algorithms were compared post hoc. Furthermore, the novel multi‐frequency ECochG algorithm produced more favorable insertion track patterns than the conventional approach using single‐frequency tone‐burst stimuli at 500 Hz. Larger amplitude drops in single‐frequency ECochG have been associated with poorer hearing preservation [19, 20, 21, 22, 23]. Multi‐frequency ECochG, we surmise, may give a more comprehensive understanding of the cochlear microenvironment during cochlear implant electrode insertion. Decreased alarms with multi‐frequency ECochG may reduce the number of compensatory adjustments during insertion, potentially decreasing cochlear trauma.

Saoji et al. described a case of a patient undergoing multi‐frequency ECochG at 500, 1000, and 2000 Hz, who showed recovery of ECochG amplitudes with real‐time monitoring and electrode retraction and repositioning [13]. This observation, together with the data presented here, suggests that multi‐frequency ECochG has the potential to increase the sensitivity and specificity of drop alarms, as ECochG drops elicited by a single frequency, chiefly reflecting the CM, may not translate to drops across all frequencies or consistently signify cochlear trauma [13]. Randomization to single‐frequency ECochG alarms being “on” during electrode insertion has been associated with decreased rates of electrode translocation from the scala tympani into the scala vestibuli compared with ECochG alarms “off.” However, this approach has not yet demonstrated significantly improved hearing preservation [20]. We hypothesize that multi‐frequency ECochG alarms may enhance hearing preservation in a large cohort of patients by reducing false or unnecessary drop alarms. This question will be addressed in our ongoing follow‐up studies.

In addition to the degree of amplitude drop, insertion track patterns in single‐frequency ECochG are possibly predictive of structural and low‐frequency hearing preservation after CI [19, 20]. With multi‐frequency ECochG, we found an increase in the number of Type A patterns—a continued increase in ECochG amplitude from the start of insertion at the round window through completion of insertion—and a reduction in Type C patterns—an amplitude at the start of insertion comparable to the amplitude at completion of insertion with the maximum reached mid‐insertion and a precipitous drop in signal thereafter [18]. This finding may indicate increased accuracy of multi‐frequency ECochG as an indication of cochlear health. When comparing single‐frequency to multi‐frequency ECochG, few participants converted from Type C to Type CC (an initial peak, followed by fluctuations then a recovery from the lowest drop amplitude) [19, 20], likely due to lack of multifrequency drop alarms provided to the surgeon to consider modifying insertion parameters. In a previous study by Harris et al., when single‐frequency ECochG drop alarms are turned on, the Type CC insertion track pattern was seen in 76% of cases (compared to 24% of cases with ECochG alarms turned off, *p* < 0.05), indicating that drop alarms may help surgeons recover ECochG signal and reduce cochlear trauma during insertion [19]. We will explore through future studies the hypothesis that using real‐time drop alarms with multi‐frequency ECochG may be associated with fewer drop alarms and preservation of cochlear structure and low‐frequency hearing during cochlear implant insertion.

Strengths of this study include its large sample size, multi‐institutional nature, and novel use of multi‐frequency ECochG. Limitations include the absence of real‐time surgeon response to drop alarms generated by the multi‐frequency ECochG algorithm and the lack of postoperative imaging and audiometric outcomes confirming structural and hearing preservation. Additionally, these data were collected using a single cochlear implant manufacturer and two distinct electrode arrays, which may limit generalizability. Ongoing investigations are evaluating hearing preservation and cochlear structural preservation with multi‐frequency ECochG compared to single‐frequency ECochG. Additionally, insertion track pattern analysis represents a simplified interpretation of inherently complex cochlear signals. While the multi‐institutional nature of the study strengthens its generalizability, variations in surgical techniques across surgeons may have influenced ECochG amplitudes and insertion tracks.

## 5 Conclusions

Multi‐frequency ECochG provides unique feedback from the cochlea and is associated with significantly fewer drop alarms and an increase in optimal insertion track patterns compared to single‐frequency ECochG. A more comprehensive understanding of the cochlear environment and parameters known to relate to functional outcomes of hearing preservation may be achieved with multi‐frequency ECochG. Future studies will focus on the direct relationship between multi‐frequency ECochG track patterns and structural/hearing preservation outcomes following CI.

## Funding

This work was supported by the Advanced Bionics, LLC.

## Conflicts of Interest

Mana Espahbodi, MD has research grant funding from Advanced Bionics and is a consultant for Cochlear Corp. Michael D. Seidman, MD is a consultant for Advanced Bionics and IotaMotion and has current funding from the Department of Defense. Esther X. Vivas is on the medical advisory board for Advanced Bionics and Medtronic. H. Jeffrey Kim, MD is a consultant for Imsmed on Arikayce. Neil S. Patel, MD, has received honoraria from Stryker Corp, has received research support from Cochlear Corp., and has functioned as a speaker for IotaMotion, MED‐EL and Zeiss. Richard Gurgel, MD has received institutional research support from Cochlear Corp, MED‐EL, Neosensory, Blackrock, and Advanced Bionics and has received travel expenses and speaker fees from MED‐EL; he is involved in medical‐legal consulting and serves on the Surgical Advisory Board for Cochlear Corp. Kanthaiah Koka is employed by Advanced Bionics. Michael S. Harris has research grant funding from Advanced Bionics and Cochlear Corp. All others authors declare no conflicts of interest.

## Data Availability

Research data are not shared.
